# A Mixed-Methods Process Evaluation of the Maastricht Work-Related Support Intervention for Healthcare Professionals in Clinical Care

**DOI:** 10.1007/s10926-024-10211-0

**Published:** 2024-06-10

**Authors:** Maarten Butink, Annelies Boonen, Tim Boymans, Vera Baadjou, Emmelie Hazelzet, Angelique de Rijk

**Affiliations:** 1 Department of Internal Medicine, Division of Rheumatology, Maastricht University Medical Centre (MUMC), P. Debyelaan 25, 6229HX Maastricht, The Netherlands; 2 Department of Social Medicine, Faculty of Health, Medicine and Life Sciences, Maastricht University, Duboisdomein 30, 6200 MD Maastricht, The Netherlands; 3 Research School Care and Public Health Research Institute (CAPHRI), Universiteitssingel 40, 6200 MD Maastricht, The Netherlands; 4 Department Orthopedic Surgery, Maastricht University Medical Centre+ (MUMC+), P. Debyelaan 25, 6229HX Maastricht, The Netherlands; 5 Department of Rehabilitation Medicine, Faculty of Health, Medicine and Life Sciences, Maastricht University, Universiteitssingel 40, 6200 MD Maastricht, The Netherlands

**Keywords:** Work participation, Chronic diseases, Intervention, Process evaluation, Clinical care, Healthcare professionals

## Abstract

**Purpose:**

To perform the process evaluation of an intervention that aims to facilitate clinical healthcare professionals (HCP) to provide Maastricht Work-Related Support (WRS) to working patients with a chronic disease.

**Methods:**

A mixed-methods approach was applied to address reach, efficacy, adoption, implementation, and maintenance (RE-AIM framework) as well as context of the Maastricht WRS intervention. Qualitative data included interviews with HCPs (*N* = 10), patients at two time points (*N* = 10 and *N* = 9), and field notes. Quantitative data included screening logbooks of HCPs, patient screening forms, and a questionnaire for patients. Content analysis or computation of frequencies was applied where applicable.

**Results:**

Twenty-eight HCPs participated in the intervention (*reach*). They had a low attitude toward providing Maastricht WRS themselves (*adoption*). During clinical consultations, they addressed work for 770 of 1,624 (47%) persons of working age. Only 57% (437/770) had paid work, of which 10% (44/437) acknowledged a current need for support. Discussing work during clinical consultations by HCPs was hindered by other medical priorities and patients not disclosing problems (*implementation*). Over time, Maastricht WRS was less consistently provided (*maintenance*). Patients reported a positive impact of the intervention, such as fitness for work (*efficacy*). *Context* (e.g., lack of urgency, priority, time, and management support) played a pivotal role in the implementation.

**Conclusion:**

This evaluation showed that HCPs had a positive attitude toward WRS in general, but their attitude toward provide Maastricht WRS themselves in daily clinical care was low. Recommendations include improving HCPs’ attitude, addressing WRS as a key policy point, and facilitating time.

**Supplementary Information:**

The online version contains supplementary material available at 10.1007/s10926-024-10211-0.

## Introduction

Persons diagnosed with a chronic disease are known to experience limitations in mental and physical functioning and restrictions in paid work participation [1, 2]. The employment rates among people with one or multiple chronic diseases continue to be lower compared to the general population (70% and 52%, respectively, compared to 74%) in Organization for Economic Co-operation and Development (OECD)-affiliated countries [3].

It has been repeatedly shown that reducing the impact of disease on health outcomes strongly improves participation [4–6]. Notwithstanding, a participation gap remains that likely reflects a complex interaction between limitations, work-related, and person-related factors, as proposed by the International Classification of Functioning, Disability and Health model [7]. Additional non-pharmacological support has been suggested, and non-pharmacological interventions have non-important to small beneficial effects [8]. However, the non-pharmacological support was often provided at advanced stages of the disease only, was not timely, and was often a one-off intervention instead of continuous and integrated support.

Studies on work participation have been conducted in populations with chronic diseases. For example, patients of working age with an inflammatory rheumatic disease are up to three times less likely to be employed than the general population and experience more problems in daily work [9–11]. Of people with inflammatory bowel disease (IBD), 9–19% experiences short-term work absences, while 19–22% faces long-term sickness absence [12]. For people with knee osteoarthritis (OA), it is estimated that sick leave costs in the Netherlands (2015–2017) amount to €26.9 million [13]. The current study focuses on these three chronic diseases, inflammatory arthritis (IA), IBD, and knee OA.

Preventing loss of paid work by continuously monitoring and supporting people with chronic disease whenever indicated seems crucial. Providing work-related support in clinical care settings can play a pivotal role, as addressed by multiple scientific reports [14, 15]. In the last decade, several interventions have been developed to support patients with work-related problems in a clinical setting [16–18]. For example, De Vries et al. developed a pathway including tools for healthcare professionals (HCPs), hired an external labor expert to support patients if indicated, focused on self-strengthening the patients, and relied highly on additional financing [18].

In these interventions, work-related support (WRS; from screening for problems to providing support) was not integrated into regular clinical care [16–18]. When problems were identified, external professionals were approached, whereas WRS should be an integral part of the clinical reasoning process of HCPs in hospitals. To this end, an intervention was incrementally developed at Maastricht University Medical Center + (Maastricht UMC +), using the Intervention Mapping protocol, for HCPs to provide WRS to working people with chronic diseases in a clinical care setting, named ‘Maastricht WRS’ [19]. The main aim was to enhance sustainable and healthy work participation by an iterative and timely intervention at points in care where patients are at specific risk for undesired (unwanted) prolonged sick leave or work disability. The Maastricht WRS intervention focusses on the behavioral change of HCPs, encouraging them to integrate support in their routine clinical care for their patients with work-related problems. It emphasizes the prevention of participation problems rather than waiting for sickness absence.

The intervention was gradually introduced at three outpatient clinics, and meanwhile developed further in response to practical experiences using an action research approach [20]. During the incremental development process, minor improvements were done (intervision was improved, tools updated, patients better informed and encouraged to discuss work with their HCP, and reminders to HCPs were improved).

The current study presents the process evaluation of the Maastricht WRS for HCPs in clinical care in terms of feasibility and acceptability, as prescribed by the IM approach [21–23]. The RE-AIM (Reach, Efficacy, Adoption, Implementation and Maintenance) process evaluation framework was used to determine its feasibility and acceptability in depth, and supplemented with the dimension ‛context,’ which refers to the barriers and facilitators at different levels (macro, meso, micro) that might influence implementation [24, 25].

## Methods

A mixed-methods process evaluation in a sequential mode was performed. Methods to collect information on the dimensions of the RE-AIM framework and ‘context’ [24, 25] will be described in chronological order (reach, adoption, implementation, maintenance, efficacy, and context) rather than following the letters of the acronym (Table 1) [26].

**Table 1:** Process evaluation dimensions applied to evaluate the Maastricht WRS

Dimensions (RE-AIM [25], unless otherwise specified)	Definition used in this study	Data collection method	Data source and sample	Time point of data collection	Indicator
Reach	The number of HCPs that participated in Maastricht WRS and their reasons	Qualitative	Interviews: sample of participating HCPs (*N* = 10)	12–24 months after the support started*	Reasons for HCPs to participate in Maastricht WRS
		Quantitative	Screening logbooks of all HCPs that provided WRS	During intervention	• Number of HCPs participating in training • Number of HCPs participating in intervision • Number of HCPs providing Maastricht WRS
Adoption	The extent to which participating HCPs are willing to provide Maastricht WRS to patients	Qualitative	Interviews: sample of participating HCPs (*N* = 10)	12–24 months after the support started*	• Attitude toward WRS in general • Attitude toward providing Maastricht WRS themselves
Implementation	The extent to which Maastricht WRS is delivered consistently and with fidelity in practice	Qualitative	Interviews: sample of participating HCPs (*N* = 10)	12–24 months after the support started*	Experiences with the intervention, training, tools, and intervision
			Interviews: sample of patients with IA (*N* = 10)	6–8 months after the intervention started (at Rheumatology)	Early experiences by patients with Maastricht WRS provided by initial HCP, nurse (low-complexity support) and/or work participation clinic (high-complexity support)
			Interviews: sample of patients (*N* = 9)	17–32 months after the support started*	Experiences by patients with receiving any component of Maastricht WRS
			Participatory observations and field notes by researcher	During intervention	Observations and field notes on the extent to which Maastricht WRS was delivered consistently and with fidelity
		Quantitative	Screening logbooks of all HCPs that provided WRS and patient screening forms	During intervention	• Proportion of patients of those scheduled for consultation and ≤ 65 years with whom work status was discussed • Proportion of patients with whom work status was discussed who mentioned work problems • Proportion of patients with whom work status was discussed who had a need for support • Proportion of patients in need of support who were referred to nurse (low complexity support) • Proportion of patients in need of support who were referred to work participation clinic (high complexity support) • Reasons for referral to work participation clinic • Types of support provided by work participation clinic
			Questionnaire for the subgroup of patients in the observational study who actually received support from nurse or work participation clinic (*N* = 20)	12 months after patient’s consultation with initial HCP	• Proportion of working patients who received advice of those who were referred to nurse/work participation clinic • Number of appointments per patient for support from nurse or work participation clinic • Satisfaction with received support from nurse or work participation clinic • Helpfulness of support from nurse or work participation clinic • Proportion of patients who received a concrete plan of those who were referred to nurse/work participation clinic • Satisfaction with formulated plan (only those who received a plan) • Performance of the plan (only those who received a plan) • Motivation to adhere to the plan (only those who received a plan) • Recommendation of Maastricht WRS to other patients
Maintenance	The extent to which Maastricht WRS is provided over time	Qualitative	Interviews: sample of participating HCPs (*N* = 10)	12–24 months after the support started*	Experiences with discussing work in follow-up consultations
			Interviews: sample of patients (*N* = 9)	17–32 months after the support started*	Experiences by patients with discussing work in follow-up consultations
		Quantitative	Questionnaire for the subgroup of patients in the observational study who actually received support from nurse or work participation clinic (*N* = 20)	12 months after patient’s consultation with their treating physician/nurse	Extent to which work was discussed in follow-up consultations
Efficacy	The impact of Maastricht WRS as experienced by patients	Qualitative	Interviews: sample of patients (*N* = 9)	17–32 months after the support started*	Proximal impact of the intervention on patients
Context [24]	Facilitators and barriers to providing Maastricht WRS, experienced by HCPs and observed by the researcher	Qualitative	Interviews: sample of participating HCPs (*N* = 10)	12–24 months after the support started*	Experiences of HCPs with facilitators and barriers to providing Maastricht WRS in the intervention’s context
			Participatory observations and field notes by researcher	During intervention	Observations and field notes on facilitators and barriers to providing Maastricht WRS in the intervention’s context

*HCP* healthcare professional, *Maastricht WRS* Maastricht Work-related Support (the specific intervention), *IA* inflammatory arthritis

^*^Providing the support started at different time points per outpatient clinic, see Fig. 2

### Intervention Description

**Fig. 1:**
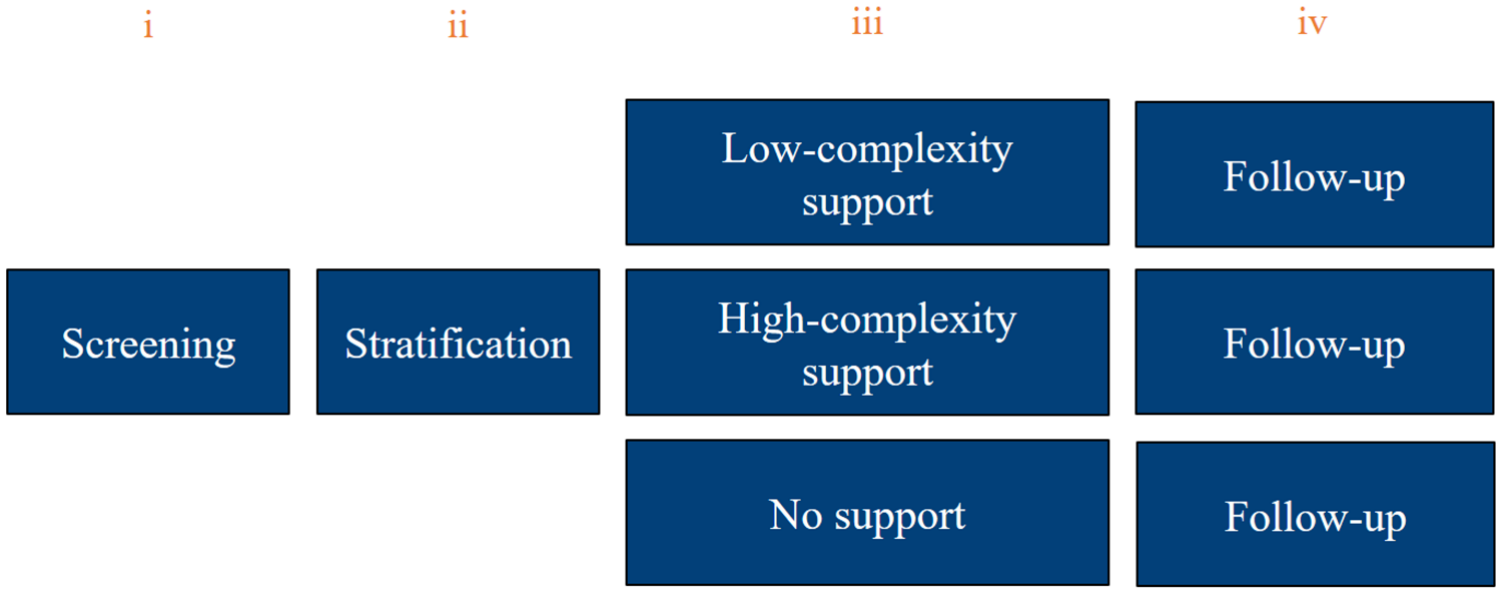
Overview of Maastricht WRS pathway and components (orange)

This intervention focused on the behavioral change of HCPs, aiming to integrate Maastricht WRS into routine clinical care, adjacent to clinical reasoning by HCPs. The Maastricht WRS care pathway combines training, tools, and intervision and is designed to achieve the behavioral change objectives required to provide the intervention alongside the routine clinical care. The care pathway comprised multiple components (Fig. 1). Screening (i) and stratification (ii) were performed in the setting of regular consultations at outpatient clinics. Support for low-complexity problems (iii) was provided by the initial HCP or a nurse after referral. In this component, the HCPs advised on non-complex problems related to work due to health. For example, HCPs address strategies for managing morning stiffness while having to work in the early hours. The HCP can offer information, adjust treatment plans, or recommend exercise therapy as part of their support. When problems in work participation were judged during screening as multifactorial and thus complex, the patient was referred to a rehabilitation physician with experience in work participation at the newly established work participation clinic (iii). For example, a self-employed roofer with severe knee osteoarthritis and limited coping abilities may benefit from a multidisciplinary approach, involving HCPs from the hospital, a rehabilitation center, as well as external organizations such as governmental organizations. Follow-up ensued (iv), irrespective of whether the patients received support.

Four supporting *tools* were developed, three for HCPs and one for patients. The first tool for HCPs included a screening card with questions to identify those patients at risk for prolonged sick leave and/or a need for support. This tool targets medical specialists or nurse specialists that run clinics independent of the medical specialist. The second targets those HCPs who deliver WRS (the nurses and rehabilitation physician) and includes five conversation cards that contain trigger questions for in-depth problem identification and stratification, while addressing four domains based on the ICF model [7]; health, work, support, and personal characteristics. The fifth conversation card invited the HCP to summarize and prioritize the need for support and to develop a care plan. The third tool for HCPs aligns with these conversation cards and consists of options for providing WRS, parallel to the domains addressed by the conversation cards (health, work, support, and personal characteristics). Examples of included options for WRS are to communicate with the employer, consider work accommodations, physiotherapy, and job coaching. The cards and map thus offered tools for advice including problem clarification by the cards and solutions to these problems by the map. The fourth tool targets patients, and contains additional information on work and health, including links to relevant (patient) websites on work and health.

*Training* was offered to HCPs to increase awareness of the necessity for the Maastricht WRS, improve clinical reasoning in relation to work participation and the use of tools. Once trained, *intervision* was organized to increase knowledge of Maastricht WRS and strengthen clinical reasoning by discussing cases with peer HCPs and experts. A comprehensive intervention description was published beforehand [19].

The intervention was gradually introduced between October 2020 and September 2021 after the introduction of Maastricht WRS and training of HCPs at three outpatient clinics at the Maastricht University Medical Center + (MUMC +), including Rheumatology (IA), Gastroenterology (IBD), and Orthopedics (knee OA). HCPs were treating physicians and nurses conducting clinical consultations.

To facilitate the introduction of the Maastricht WRS and to allow a smooth informed consent procedure for the planned structured evaluation, all eligible patients were informed by letter, one week before the planned clinical consultation, about a care initiative and possibility to participate in a questionnaire survey on quality of care. Patients were eligible if they were diagnosed with IA, IBD, or knee OA, of working age (18–65 years), and had paid work (≥ 12 h per week, including self-employment and working for a temporary work agency). Patients were excluded if they had a short life expectancy, were a full-time student, were on prolonged sick leave (≥ three months), had a labor dispute with their employer, or were unable to read or understand the Dutch language.

**Fig. 2:**
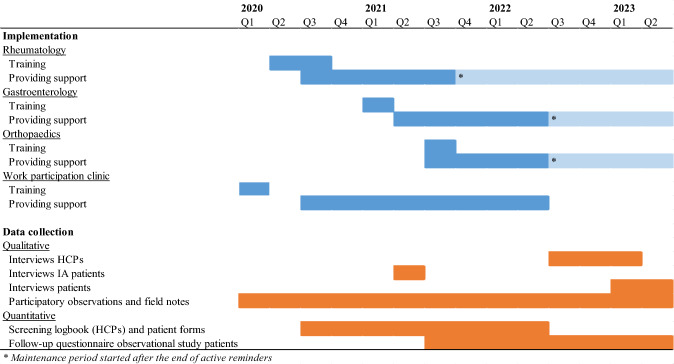
Implementation and data collection timeline of the Maastricht WRS intervention

At the start of daily clinical consultations, the HCPs were given a screening logbook of scheduled patients who received the letter. The screening logbook was a reminder to provide Maastricht WRS during clinical consultations, but at the same time a logbook for data collection in which HCPs had to note whether the patient with paid work (a) had experienced sick leave in the past six months, (b) experienced problems in work due to health, (c) had a need for support, and (d) were willing or not to participate in an observational study about health and work. After 12–18 months, no more patient letters were sent out and no more screening logbooks were provided to HCPs, as we expected to have already a sufficient number of persons in the parallel observational study (not published yet) and HCPs would have sufficient self-efficacy (Fig. 2). Active reminders and inclusion of patients in an observational questionnaire study stopped in December 2021 (Rheumatology) and May 2022 (Gastroenterology/Orthopedics).

When proposing the above approach to Orthopedics, it turned out it was not possible for their HCPs to perform the screening due to time constraints. The project group and HCPs from Orthopedics decided patients would receive a patient screening form at home attached to the pre-consultation information letter to (a) indicate whether they experienced problems in work participation, including sick leave, (b) had a need for support, and (c) were willing or not to participate in an observational study about health and work. Patients were asked to handover the completed form to their HCP during the clinical consultation, who could then refer the patient with need for support to the work participation clinic, independent of stratification into complexity.

### Data Collection

Data to inform the RE-AIM and context dimensions were retrieved from multiple sources (Table 1; Fig. 2).

#### Qualitative Data

Four groups representing relevant persons or stages of Maastricht WRS were interviewed.

First, interviews with a purposive sample of HCPs (*N* = 9/28) who participated in screening and stratification (physicians and nurses from Rheumatology and Gastroenterology), low-complexity support (physicians and nurses from Rheumatology and Gastroenterology), and referral without active screening (physicians from Orthopedics) were conducted 12–24 months after the start of the intervention.

Second, an interview with the rehabilitation physician from the work participation clinic was conducted 24 months after the start of the intervention.

Topics in these interviews with HCPs (including the rehabilitation physician) were reasons to provide the Maastricht WRS, experiences with the care pathway and intervention components, training, tools, intervision, individual perception of behavioral change, and facilitators and barriers to providing Maastricht WRS.

Third, a random sample of patients participating in the observational study was invited for an interview in the early phase of the intervention. At that time (7–9 months after the start of the intervention), the intervention had only been implemented in the Rheumatology clinic; therefore, only IA patients (*N* = 10/106) who had participated in the observational study till then were considered. Topics comprised experiences with their HCP asking about work and, if referred for additional support, their experiences with low- or high-complexity support.

Fourth, a second patient sample (*N* = 9/285) who had participated in the observational study was selected and interviewed between 19 and 31 months after the intervention started. This sample was purposively selected to get a proportional distribution of disease (IA, IBD, knee OA), type of support received (only discussing work with their initial HCP; low-complexity support from a nurse; or high-complexity support at the work participation clinic), gender, and age (≤ 50 vs. > 51). Topics comprised expectations of Maastricht WRS, experiences of discussing work with their initial HCP, support received, impact experienced, and follow-up of the support provided.

All interviews (HCP and patients) were conducted either online or in the hospital setting, were recorded with Microsoft Teams, and lasted 14–49 min.

Additionally, participatory observations regarding all aspects of the process evaluation were collected by the first author (MB) as project coordinator, supplemented with field notes on conspicuity or deviations from the intervention as intended (Table 1; Fig. 2).

#### Quantitative Data

First, the number of patient letters sent to patients combined with the screening logbooks for HCPs (Rheumatology/Gastroenterology) and patient screening forms (Orthopedics) provided quantitative data on work participation, sick leave, restrictions experienced in participation, and need for support.

Second, data from the 12-month questionnaire of patients participating in the observational study who had received additional care from the nurse (low-complexity support) or the work participation clinic (high-complexity support) (*N* = 20/27) provided data to evaluate the Maastricht WRS in terms of satisfaction and helpfulness of the support and explored the extent to which work was discussed in follow-up consultations (Table 1).

### Data Analysis

Interview data were transcribed ad verbatim. Interview data and field notes were analyzed according to directed content analysis by MB in three steps: 1. coding interview parts into the process evaluation dimensions, 2. clustering codes on low abstraction level, and 3. identifying and defining themes per process evaluation dimension [27]. ATLAS.ti software (version 23.0.7) was used for the coding and analysis.

For all quantitative data (screening logbooks, patient screening forms, and questionnaires), descriptive analyses were performed to compute numbers, means (SD), and frequencies. Analyses were performed in SPSS version 27 and Excel version 2016.

### Ethical Statement

The protocol of the evaluation of the Maastricht WRS intervention was reviewed by the MUMC + Medical Ethics Committee (METC2021-3001). It was agreed that the Medical Research Involving Human Subjects Act did not apply. All participants in the qualitative or qualitative evaluation provided written informed consent.

## Results

### Reach

A total of 28 HCPs were informed about the intervention’s background and only the HCPs from Rheumatology and Gastroenterology (20/28, 71%) were trained as planned. As an alternative to the time-consuming training, HCPs in Orthopedics were instructed during weekly briefings before clinical consultations. Despite recurrent invitations to all 28 HCPs, only a minority of HCPs participated in intervision sessions (2/28, 7%) (Online resource 1).

The same 28 informed HCPs participated in the Maastricht WRS, performed screening and stratification, provided additional support, and/or referred patients who indicated a need for WRS (the number of patients reached is addressed under ‘Implementation’). Interviewed HCPs indicated they participated in the Maastricht WRS intervention because of a higher-level decision, while others felt intrinsically motivated.

*“Let me be very honest that the main reason was that the department head said we were going to do that. So, out of myself I hadn't started doing this.” (HCP 5 screening patients)*

*“… also because stress at work, desire, or need to work, but not being able to work because of diarrhea or fatigue, still has a very big psychological burden on patients which again may lead to worse outcome of the disease.” (HCP 4 screening patients)*

### Adoption

Interviewed HCPs expressed a positive attitude toward WRS in general. However, their attitude to provide the Maastricht WRS themselves was considerably lower. HCPs mentioned some barriers that hindered the discussion of work-related problems and providing support, including limited priority due to other medical topics, time constraints, and the topic of work not yet being standard in their routine clinical care.

*“Yes, but not systematically enough and it also does often drop down the list, given limited consultation time.” (HCP 7 screening patients)*

### Implementation

#### Implementation of Training, Tools, and Intervision

Of those HCPs who were trained (from Rheumatology and Gastroenterology), interviewed HCPs described the *training sessions* as useful in preparing them to provide Maastricht WRS. Participatory observations and field notes showed that the training sessions were adapted on-site, as HCPs were not inclined to engage in role-playing exercises for practice.

HCPs expressed that the training increased their awareness of the importance of work participation, resulted in more knowledge, and increased their self-efficacy to support patients.

*“And you start applying those things that you learn, and then you notice that it does pay off in the end. So I definitely think it adds value.” (HCP 3 screening patients)*

HCPs also emphasized that the training should be repeated, preferably after the first lot of patients is seen, to evaluate and improve the support they provide.

*“How did it go? Was that a senseless question I asked? Did I send the patient to the wrong professional? And so you can actually improve the support.” (HCP 1 providing low-complex support)*

HCPs indicated the *tools* were not systematically used during clinical consultations; one group of HCPs did not use the tools at all, while others used the tools only to prepare for consultations. The content of the tools was familiar to a group of HCPs, and one HCP admitted the tools forced her to follow a format in her way of conducting patient consultations. HCPs mentioned that they did not appreciate multiple separate tools and expressed the need for only one comprehensive tool (one-paper sized and preferably also digitally available on the hospital website).

*“The tools do have a lot of information. A very short flowchart to screen and support patients would maybe work better than all those separate tools, because then you’re through it faster.” (HCP 3 screening patients)*

Finally, a minority of HCPs (2/28, 7%) attended an *intervision* session (Online resource 1). Non-attending HCPs had postponed attendance repeatedly and/or mentioned lack of time. Participants regarded intervision as particularly useful. They indicated they preferred to discuss case studies and suggested organizing intervision sessions more frequently. However, participatory observations and field notes on this topic revealed the participating HCPs mainly relied on and expected input from invited experts; their active contribution in sharing experiences, submitting cases, and participating in role playing was limited.

#### Implementation of Screening and Stratification

##### Quantitative

In 770 of 1624 patients (47%) scheduled for consultations who were 18–65 years old and potentially working, work status was discussed by HCPs (733 from Rheumatology and Gastroenterology) or by the patients themselves on screening forms (37 from Orthopedics). Among non-working patients (333/770, 43%), work disability (including full, partial, and young disabled) appeared as the most frequent reason (33%). Of those with paid work (437/770, 57%), restrictions in work participation were identified by HCPs during consultations or by the patients themselves on the screening forms (Table 2).

**Table 2:** Number of patients and their characteristics included in the Maastricht WRS intervention, total and per outpatient clinic separately

	Total	Rheumatology	Gastroenterology	Orthopedics
Patients less than 65 years old informed by letter before the consultation	1624	944	496	184
Mean age (SD)	48.6 (12.4)	52.4 (11.4)	42.9 (13.7)	44.8 (14.1)
Female (%)	58.8%	60.0%	59.8%	50.0%
Work status collected^a^	770 (47.4% of 1624)	546 (57.8% of 944)	187 (37.7% of 496)	37 (20.1% of 184)
Mean age (SD)	48.2 (11.5)	51.3 (10.9)	39.3 (12.8)	47.7 (13.3)
Female (%)	57.7%	59.8%	52.9%	51.4%
Not working or working < 12 h p/w	333 (43.2% of 770)	261 (47.8% of 546)	63 (33.7% of 187)	9 (24.3% of 37)
Reason non-collected	169 (50.7% of 333)	144 (55.1% of 261)	18 (28.6% of 63)	8 (88.8% of 9)
Due to full work disability	90 (27.0% of 333)	76 (30.3% of 261)	14 (22.2% of 63)	1 (11.1% of 9)
Due to partial work disability	19 (5.7% of 333)	10 (3.8% of 261)	9 (14.3% of 63)	NA^b^
Due to unemployment/job hunting	23 (6.9% of 333)	9 (3.4% of 261)	14 (22.2% of 63)	NA^b^
Due to full-time student	16 (4.8% of 333)	9 (3.4% of 261)	7 (11.1% of 63)	NA^b^
Due to job loss due to COVID-19	8 (2.4% of 333)	8 (3.1% of 261)	1 (1.6% of 63)	NA^b^
Due to early retirement	4 (1.2% of 333)	4 (1.5% of 261)	0	NA^b^
Due to Wajong benefit^c^	1 (0.3% of 333)	1 (0.3% of 261)	0	NA^b^
Working ≥ 12 h p/w (and eligible for Maastricht WRS)	437 (56.8% of 770)	285 (52.2% of 546)	124 (66.3% of 187)	28 (75.7% of 37)
Working patients indicating restrictions in work participation*	437	285	124	28
Mean age (SD)	46.9 (12.6)	50.4 (10.5)	38.9 (13.1)	46.8 (13.5)
Female (%)	57.2%	61.8%	47.6%	53.6%
Type of restriction in work participation:				
Sickness absence in past six months	49 (11.2% of 437)	23 (8.1% of 285)	26 (21.0% of 124)	^d^
Problems in work due to health	97 (22.2% of 437)	53 (19.6% of 285)	23 (19.5% of 124)	21 (75.0% of 28)^d^
Need for support	44 (10.0% of 437)	15 (5.3% of 285)	10 (8.1% of 124)	19 (67.9% of 28)
Maastricht WRS of patients indicating a need for support	44	15	10	19
Mean age (SD)	47.0 (13.3)	52.0 (9.4)	39.8 (15.6)	46.8 (13.5)
Female (%)	50.0%	60.0%	50.0%	42.1%
No referral^e^	10 (22.7% of 44)	4 (26.7% of 15)	0	6 (31.6% of 19)
Referral to nurse (low-complexity)	9 (20.5% of 44)	6 (40.0% of 15)	3 (30.0% of 10)	NA^f^
Referral to work participation clinic (high-complexity)	18 (40.9% of 44)	5 (33.3% of 15)	6 (60.0% of 10)	7 (36.8% of 19)
Referral to work participation clinic canceled by patient	7 (38.9% of 18)	0	1 (16.7% of 6)	6 (85.7% of 7)

^a^Work status and restrictions in work participation were discussed during consultations (Rheumatology and Gastroenterology) or collected via patient screening forms (Orthopedics)

^b^Not questioned on patient screening forms (Orthopedics) to prevent filling in the forms from becoming too time consuming

^c^Wajong benefit is granted to an insured person if this person became work disabled before their eighteenth birthday and remains so on their eighteenth birthday

^d^Both questions were combined into one on the patient screening form (only for Orthopedics), and numbers were included in the item ‘problems in work due to health’

^e^Reasons for not referring were HCPs themselves supported patients with their work problems and further referral was not necessary, or patients did not want an additional appointment to discuss work problems, or it was unclear to patients what the consultation was about

^f^No nurse to provide low complexity support was available at this outpatient clinic

Sickness absence in the last six months and difficulties in ability to work due to their health were experienced by 11% (49/437) and 22% (97/437), respectively. A need for support was indicated in 10% of screened patients.

The need for support was substantially higher (68%) in Orthopedics, where patients indicated this on the screening form themselves, probably because only those who wished to discuss work remembered to hand in the completed form to their HCP. In Rheumatology and Gastroenterology, these numbers were lower (5–8%), when asked by the patient’s HCP during face-to-face consultations (Table 2).

#### Implementation of Screening

##### Qualitative

HCPs expressed a strong dependence on the screening logbooks to remind them to discuss work. Even though they had the screening logbooks, some HCPs mentioned that they were only reminded to discuss work when the patient initiated the conversation (prompted by the information letter received at home).

Some HCPs expressed concern about patients who felt uncomfortable disclosing these problems around work to their HCP and therefore risked underestimating health-related problems at paid work. This concern extends not only to work-related issues but also to other relevant life domains.

*“The people who are not open about it, I think that is a significant obstacle. Those people who find it difficult to communicate about their problems.” (HCP 10 screening patients)*

A group of interviewed patients (Rheumatology) reported that their HCP always started a conversation on issues with paid work and in hindsight they now recognize that they did not fully appreciate its value back then.

*“So the rheumatologist and the rheumatology nurse were always paying attention to the topic of work anyway, to point it out to me. Only back then, that didn’t always want to sink in.” (Patient 4 screened by its HCP)*

#### Implementation of Stratification

##### Qualitative

HCPs indicated that they were able to stratify patients, partly due to their experience with clinical reasoning. If a patient did not provide any indications of problems, HCPs mentioned that stratification did not take place.

*“I assume that if I ask the question, how are things at work, are there any problems? And if someone says, yes, things are going well. Then I'm not going to ask any further in-depth questions.” (HCP 7 screening patients)*

One HCP indicated that her previous experiences also played a role in stratifying and referring, considering that some past patients did not follow previously given advice. It was also expressed that these patients who did not follow previously given advice might be the ones who required more attention.

*“Then you weigh up for yourself whether it makes sense with this patient to go into this even further and insist on it, or do you think, never mind, I’ve explained things here so many times and offered help and it doesn’t happen anyway. [...] probably those are the patients who need extra attention, who have resistance and those are probably the patients we don’t have enough time for then.” (HCP 5 screening patients)*

HCPs mentioned that, in addition to stratification, limited time also played a role in referring patients to, for example, the nurse.

*“If I notice that there is an issue then, I try to discuss that extra. And otherwise, I have people come back to the nurse, for example, to discuss it in more detail, who also take half an hour or an hour to do so.” (HCP 4 screening patients)*

#### Implementation of Referring Patients with Low-Complexity Problems

##### Quantitative

On average, 21% (range 30–40%) of patients was referred for low-complexity support (Table 2; Online resource 2). No nurse was available to provide this support in the Orthopedic clinic (and HCPs were allowed to refer to the work participation clinic independent of stratification into complexity). In Rheumatology, six patients with a need for support (6/15, 40%) and in Gastroenterology, three patients (3/10, 30%) were referred from their HCP to the nurse for low-complexity support (Table 2).

#### Implementation of Providing Support for Low-Complexity Problems

##### Qualitative

Nurses providing low-complexity support indicated that they listened, provided support and advice, and, if indicated, referred patients to their own occupational physician (if they had one) or the work participation clinic for more complex problems. Nurses mentioned that they missed the opportunity to refer patients to information on work and health on the hospital’s website.

Concern was expressed by a group of nurses about patients with limited health literacy, who are less able to face problems (in work), and those who do not want to rely on others and therefore do not ask for help.

*“Because that’s often what you find people running into. Because asking for help actually means they are dependent, and no one wants to be dependent. And that’s where you do notice bottlenecks. Added to that, you then of course have the patients what are not so health conscious. That's another problem, of course.”(HCP 3 providing low-complexity support)*

Interviewed patients expressed different views on support received from the nurse; a group of them received suggestions and advice, while others missed aspects from their nurse.

*“But I was actually missing the concrete advice or tips or handles or information to figure something out or maybe coordinate with an employer or other things, more, more practical advice.” (Patient 2 referred for low-complexity support)*

Upon further questioning by the interviewer, patients indicated that they had not received the flyer from their HCP and thus the experience with this tool could not be explored further among the interviewed patients.

#### Implementation of Providing Support for High-Complexity Problems

##### Quantitative

On average within the three patient groups, 41% of patients with a need for support was referred to the work participation clinic for *high-complexity support* (Table 2; Online resource 2). A higher percentage of patients referred (60%) were observed from Gastroenterology, compared to Rheumatology (33%) and Orthopedics (37%).

HCPs referred patients to the work participation clinic who were experiencing physical or mental problems in relation to work (mentioned in 11/18 referral letters, 61%). They requested a ‘general advice’ without specification (6/18, 33%) or no reason was mentioned (1/18, 6%). Various (combined) types of support were provided to those who had an appointment at the work participation clinic (Online resource 3).

A substantial proportion of referred patients (7/18, 39%) canceled the appointment at the work participation clinic, with the majority of them from Orthopedics (6/7, 86%).

#### Implementation of Providing Support for High-Complexity Problems

##### Qualitative

The rehabilitation physician running the work participation clinic indicated that she expected more patients to be referred to this clinic but also mentioned that some referred patients were not necessarily of high complexity. However, for those properly stratified, this HCP indicated that the work participation clinic was valuable for them. The lack of one integrated electronic patient file (which was separate at the time of the study) to communicate with the patient’s initial HCP was perceived as a barrier. Suggestions for improvement were to facilitate evening consultations at the work participation clinic after the patients’ working days and organize a physical outpatient clinic completely dedicated to work to increase visibility for both patients and screening HCPs.

Patients expressed satisfaction with the work participation clinic, but a group also indicated a lack of clarity about the purpose and possibilities this clinic could offer in relation to work participation. They were given advice or follow-up therapy, which was appreciated. One interviewed patient indicated that he did not realize at the time of consultation that he was at risk for work participation problems and in hindsight feels that he would have liked to have been made aware of this by HCPs earlier.

*“It was actually left to me to do something with it. So maybe the conversation could have been a bit more pointed out, like, listen: ‛You think, you may think it’s all still going, but just take it from me that it will bother you in the long run.’ That could maybe have been slightly emphasized that, that could indeed be a concern over time, or maybe already at that moment.” (Patient 1 referred for high complexity support)*

A group of interviewed patients who canceled the appointment indicated an additional appointment was actually not necessary, and others indicated the value of the work participation clinic was insufficiently explained.

### Maintenance

Over time, patients seen by the nurse or at the work participation clinic reported that the extent to which work was discussed in follow-up consultations with their initial HCP was low (Online resource 4). HCPs indicated that no longer receiving the screening logbook (that also acted as a reminder) led to them forgetting to discuss the topic of work and recommended incorporating reminders in the electronic patient file to discuss work participation.

*“But you also notice as soon as that* [reminders by screening logbook] *stops happening, you go back to your daily routine. So that it just fades away or gets forgotten.” (HCP 6 screening patients)*

The rehabilitation physician running the work participation clinic mentioned it was a barrier in the follow-up that she was not informed by the initial HCPs about how the support provided was progressing and the current restrictions in work participation of patients who attended the work participation clinic.

*“And also the patient, I sent one or two to a first-line psychologist, and referred back to a rheumatologist, in this case. […] I only now realize myself that I don’t know if it went well what we had thought of together, because, yes, that’s up to the treating physician/nurse again.” (HCP 2 at the work participation clinic)*

Patients revealed that work was no longer discussed during follow-up consultations unless they addressed the topic of work themselves, reflected by the low mean scores for discussing work during follow-up (Online resource 4). Other patients explained they did not bring up work-related topics as they perceived rush and a lack of time at the outpatient clinic.

*“There is always a certain rush behind it, so the appointments always run over, [...] but I do always come in there with the idea of they are already running behind and they don’t have much time. I actually don’t allow myself the time to let the conversation last longer.” (Patient 1 screened by its HCP)*

### Efficacy

Patients described the proximal impact of Maastricht WRS in general as positive and mentioned examples, such as improved fitness for work.

“*But I have to say, when I went to the work participation clinic and when I actually finished, I did find that I was physically a bit better for work, that anyway.” (Patient 4 referred for high complexity support)*

Specifically low- and high-complexity support as well as support provided by experts outside the hospital (after referral to, e.g., an occupational therapist or physiotherapist) were described as helpful by the patients, because of insights gained or practical work-related advice received, such as regarding work-life balance, work accommodations, and stress relief.

*“He* [nurse] *did give hope. He was someone who did want to cheer you up and make sure that there was still a horizon somewhere.” (Patient 2 referred for low complexity support)*

### Context

Based on the participatory observations, field notes, and interviewed HCPs, context played a role throughout the entire implementation process. Several barriers and facilitators in the context were identified (at three different levels), which may also explain the variations in implementation between the outpatient clinics.

At the macro level (national), the COVID-19 pandemic was regarded by HCPs as a challenge to integrate discussing work into routine clinical care, as many consultations were canceled. In addition, the competing priorities in a setting with staff shortages was mentioned as a barrier.

*“It’s the pressure, it’s the stress, it’s the staff shortage within care.” (HCP 1 providing low complex support)*

At the meso level (hospital organization), HCPs mentioned as a barrier the lack of hospital-wide support from the management to emphasize the importance of incorporating WRS into routine clinical care. Increasing the visibility of Maastricht WRS for both patients and HCPs within the hospital and expanding the intervention to other patient groups were mentioned as facilitators.

*“That if the hospital finds that important, that they should help find ways to fund it. [...] I think it is allowed nationwide, with employer bodies, or UWV* [Institute for Employee Insurance]*, it is a joint responsibility, I think. [...] Then you’ll have the discussion: is ‘work’ healthcare money, yes or no, or from what other funding? And that’s where I expect a role for the hospital board, in that high-level discussion.” (HCP 2 at the work participation clinic)*

At the micro level (intervention at the different outpatient clinics), an increase in reminders for discussing work was mentioned as a facilitator for providing Maastricht WRS. A group of HCPs cited lack of social norms and urgency to discuss work among colleagues as barriers. In addition to context influencing the implementation process, the extent to which context played a role varied among the outpatient clinics.

## Discussion

The current study addresses the implementation process of the Maastricht WRS intervention for HCPs in a clinical care setting. The feasibility and acceptability of the intervention were addressed using the RE-AIM and context dimensions [24, 25]. In general, HCPs had a positive attitude toward WRS in general, but their attitude toward providing Maastricht WRS themselves in daily clinical care was considerably lower. Implementation was seriously impaired, reflected by work only being discussed in 47% of patient consultations. A lack of urgency, priority, time, and limited management support hindered implementation of Maastricht WRS.

**Fig. 3:**
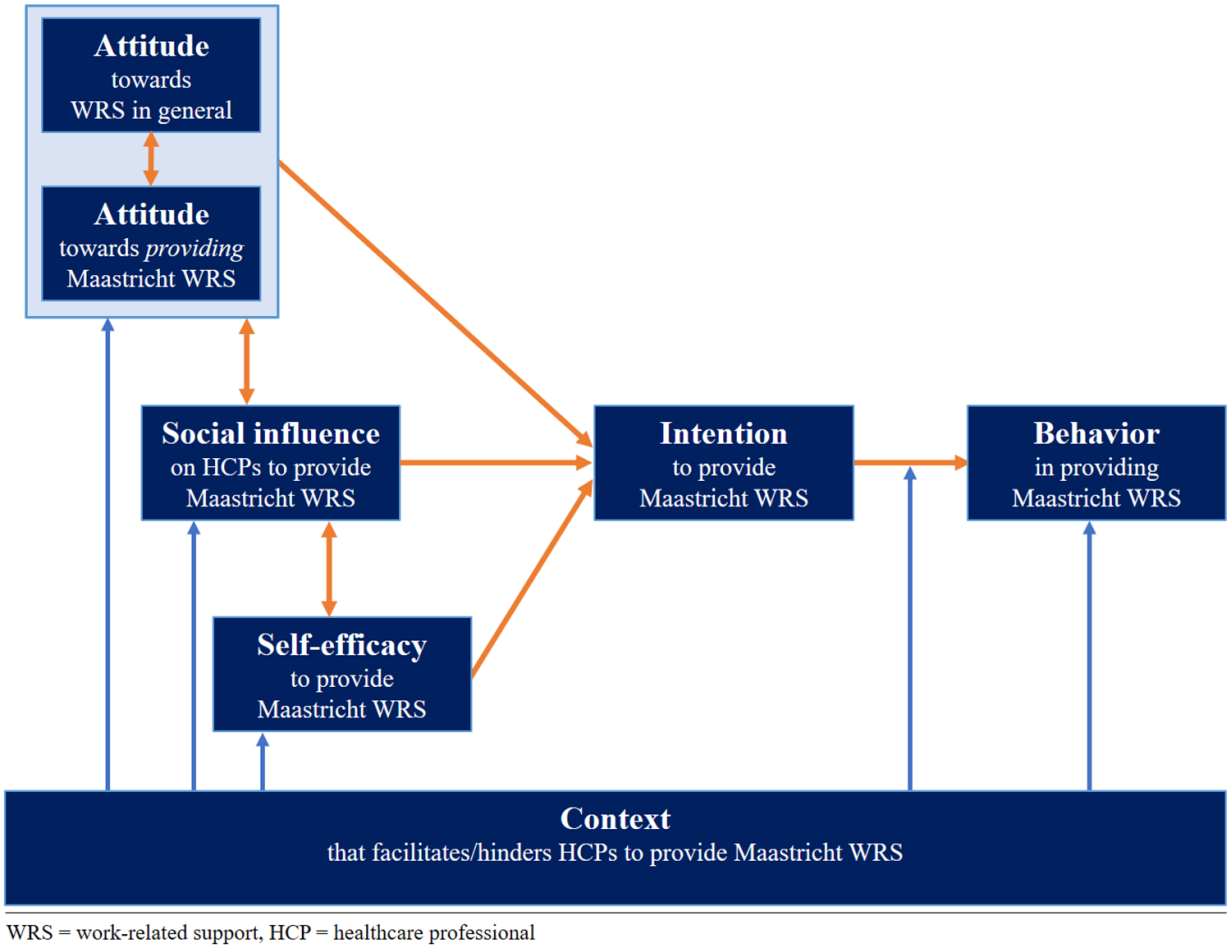
Consecutive phases required to achieve behavioral change among HCPs to provide Maastricht WRS, adopted from De Vries et al. [29] and as observed in the process evaluation

Overall, there seems to be an implementation failure due to the underutilization of the Maastricht WRS intervention. HCPs expressed a positive attitude toward WRS in general, and initially, the members of the project group assumed that this attitude would result in actual integration of the Maastricht WRS intervention in daily clinical care (**adoption**). A potential explanation can be found in the intervention’s development, where the change objectives concerning attitude (a primary determinant for other behavioral determinants and ultimately leading to behavior change) appeared to focus excessively on HCPs’ general attitude toward WRS, rather than their own behavior in actually providing Maastricht WRS to their patients [19]. Stagnation in the consecutive phases of behavioral change of HCPs occurred because they were not sufficiently open to providing Maastricht WRS (Fig. 3). A positive attitude toward WRS in general can, at best, enhance one’s attitude toward providing Maastricht WRS themselves. It certainly does not have any direct effect on intention. As a result, subsequent phases of behavioral change (determined by social influence and self-efficacy determinants) may also not be adequately addressed through training, tools, and intervision aimed at providing support [28].

Additionally, not every HCP attended the training sessions, and training participants did not make optimal use of the training sessions, such as participating in role-play or sharing cases. Additionally, despite repeated invitations, there was limited participation in intervision, and the use of tools was low, suggesting attrition of HCPs’ motivation to provide Maastricht WRS (**reach**).

Regarding the proportion of eligible patients with whom work status was discussed (47%), one might question whether this percentage is high or low and what it might have been without this intervention, especially considering that discussing work-related problems is advocated in the RA professional guideline [30]. The proportion of screened patients with work-related problems was particularly high in Orthopedics, possibly explained by patients only handing in their patient screening forms when problems were present, potentially resulting in selection bias due to self-screening. In this patient group, the self-reported need for support was also higher compared to the other two outpatient clinics. Pertinently, self-screening, as performed in Orthopedics, may not identify the truly vulnerable individuals (**implementation**).

Despite HCPs in Orthopedics not following the training as intended, nor the intervision, patients with work-related problems were still identified. Out of the 44 patients with a need for support, 27 patients received support from the nurse or work participation clinic. It was noteworthy that patients (mostly from Orthopedics) canceled their appointments with the work participation clinic due to a lack of clarity about the support trajectory. Training and intervision for HCPs could play a role in better informing patients about possible support trajectories.

Patients highlighted the impact of the support provided in the Maastricht WRS intervention, which included benefits related to their ability to engage effectively in their work, such as improved fitness for work (**efficacy**).

Regarding the underutilization of the Maastricht WRS, explanations can be found within the **context** of the intervention (facilitators and barriers to providing Maastricht WRS as experienced by HCPs and observed by the researcher). The context matters and plays a role both at the societal level [9] and within the setting of this WRS intervention. First, the implementation of the intervention was influenced by general staff shortages in healthcare, and changes in daily care due to COVID-19 may have played a role (macro level). Second, the limited hospital-wide support by the management also seems to be a contributing factor for all outpatient clinics (meso level). Finally, the different approaches of the care pathway and screening at the outpatient clinics could explain the variations in the number of patients. Additionally, HCPs mentioned that the absence of screening logbooks hindered the **maintenance** of discussions about work. Thus, it appears that behavioral change was not achieved, especially considering that social norms (addressing and encouraging each other) had not changed. It should be noted that the extent to which context had an influence differed by outpatient clinic, depending on (human) resources (micro level).

Overall, the Maastricht WRS intervention showed limited implementation among HCPs, mainly due to a low attitude. In a Dutch intervention by De Vries et al., a WRS program for people with a kidney disease was tested among HCPs [18]. This intervention’s implementation was rated with an overall score of 8.4 (out of 10) by participating HCPs. Particularly, the integration of screening into the electronic patient file was valued as they provided a reminder for HCPs to pay attention to work [18]. In the Maastricht WRS, the electronic patient system had not yet been equipped to remind HCPs to discuss work and provide WRS.

The study of De Vries et al. emphasizes that discussing work-related topics is not (yet) a routine during clinical consultations [18]. A recent scoping review estimated that discussions about work were initiated by HCPs, patients, or both, in 15–52% of clinical consultations. Our finding of 47% might point at a modest implementation success [31].

While we provided training sessions and tools to HCPs, our study indicates that this alone may not be adequate to facilitate and ensure behavioral change. Similar results were observed in the study by Jensen et al., to implement the Conversational Health Literacy Assessment Tool (CHAT) tool for HCPs, with the aim to assess individual health literacy needs in various clinical settings in Denmark [32]. Although the CHAT-tool was valued feasible, there were still important considerations for implementation (for example to implement the tools broad-scale or organizational wide instead of only within a small-scale project) [32].

### Methodological Considerations

In this study, the inclusion of different HCPs and patient samples was a strength. However, the samples were small, which limits internal and external validity of the results. Also, the numbers were too low to demonstrate significant differences in statistical tests. The study exclusively concentrated on participating outpatient clinics within one university medical center, which also hinders its generalizability. However, the data triangulation due to multiple data sources (interviews, participatory observations and field notes, questionnaires, screening logbooks, and patient screening forms) improved the internal validity of the results.

The RE-AIM framework lacks contextual factors, which prompted the authors to include this dimension from Steckler and Linnan in the current evaluation [24, 25]. The predetermined order of the RE-AIM framework proved to be a limiting factor in crystallizing the implementation problem, resulting in a chronologic ordering of dimensions.

### Recommendations for Research

In future evaluations using the RE-AIM framework, careful consideration should be given to how context should be included and studied.

Research is needed to explore more specifically the attitude of HCPs regarding providing WRS interventions themselves (in contrast to their attitude toward WRS in general). When there is a limited attitude, addressing self-efficacy and social norms becomes challenging, which calls for further research in this regard.

Finally, collaborative efforts can be made on a larger scale to conduct (cost-effectiveness) evaluation studies on similar interventions with other academic hospitals in the Netherlands (e.g., Radboud UMC, UMC Groningen). Evaluating the type of support needed, the support that was provided, and the effects observed on work participation outcomes can result in better understanding.

### Recommendations for Practice

First, the attitude of HCPs toward the specific Maastricht WRS should be improved. Appropriate evidence-based methods to change this attitude include anticipated regret, new arguments, and persuasive communication [23]. Only when their attitude to their own behavior is sufficiently established can efforts be made to strengthen self-efficacy and social norms (Fig. 3).

Second, consideration should be given to facilitating time for WRS in clinical care. Government, healthcare insurers, and hospital management can play a prominent role in this by regarding WRS as a fundamental focus of prevention and healthcare policy, and allocating time for HCPs to provide WRS. It is important for HCPs to feel supported to provide WRS, in an era of staff shortages, where only the most essential medical priorities can be discussed.

Third, the intervention has mainly been carried out by early adopters. It is relevant within the hospital to assess to what extent this intervention can be disseminated to its other outpatient clinics to increase the urgency for WRS and capacity to provide support, and to study effectiveness.

Finally, the intervention has the potential to become more integrated into daily clinical care. Reminders and patient-related work data should be included in electronic patient files, training, and intervision on WRS should be incorporated into the basic training curricula in hospitals and medical education, and HCPs involved in WRS (both within and outside the hospital) should be facilitated to collaborate closely.

## Conclusion

The implementation evaluation of the Maastricht WRS intervention showed that HCPs had a positive attitude toward WRS in general. However, their attitude to provide Maastricht WRS themselves appeared low. Recommendations to improve the intervention include improving HCPs’ attitude, addressing WRS as a key policy point, and facilitating time to improve integration of the intervention in daily clinical care.

The findings of this study contribute to the ongoing development of interventions aimed at supporting people with chronic diseases in their work participation, ultimately enhancing their overall well-being and quality of life.

## Supplementary Information

Below is the link to the electronic supplementary material.

Supplementary file1 (PDF 402 KB)

## Data Availability

Not applicable.
