# Supplementary material for: A Mixed-Methods Process Evaluation of the Maastricht Work-Related Support Intervention for Healthcare Professionals in Clinical Care
Source: J Occup Rehabil. 2024 Jun 10;35(2):374–89. doi: 10.1007/s10926-024-10211-0 (PMC12089242; doi:10.1007/s10926-024-10211-0)
Supplement: Supplementary file 1 — Supplementary file1 (PDF 402 KB) [file 10926_2024_10211_MOESM1_ESM.pdf]

Supplementary Information

A mixed-methods process evaluation of the Maastricht work-related support intervention in clinical care for healthcare professionals in clinical care

Butink, M., Boonen, A., Boymans, T., Baadjou, V., Hazelzet, E., & De Rijk, A.

Journal of Occupational Rehabilitation

Correspondence

Correspondence should be addressed to M.H.P. Butink, Department of Internal Medicine, Division of Rheumatology, Maastricht University Medical Centre+, P. Debyelaan 25, 6229 HX, Maastricht, the Netherlands (e-mail: m.butink@maastrichtuniversity.nl), +31(0)43-3875026

Content

Online resource 1. HCPs participating in training and intervision sessions 2

Online resource 2. Bubble figure of number of patients in the Maastricht WRS intervention (from approaching patients to providing support) 3

Online resource 3. Types of support provided at the work participation clinic 4

Online resource 4. Evaluation by patients in the observational study that received low- or high-complexity support 5

Online resource 1. HCPs participating in training and intervision sessions

	**HCPs entitled to provide Maastricht WRS**	**Participated in training**			**Participated in intervision**		
		Total	Physician*	Nurse	Total	Physician*	Nurse
Rheumatology	13	13	11	2	2	1	1
Gastroenterology	6	6	4	2	0	0	0
Orthopedics	8	0	0	0	0	0	0
Rehabilitation	1	1	1	0	0	0	0
Total	28	20	16	4	2	1	1

*Including physicians in training

Online resource 2. Bubble figure of number of patients in the Maastricht WRS intervention (from approaching patients to providing support)

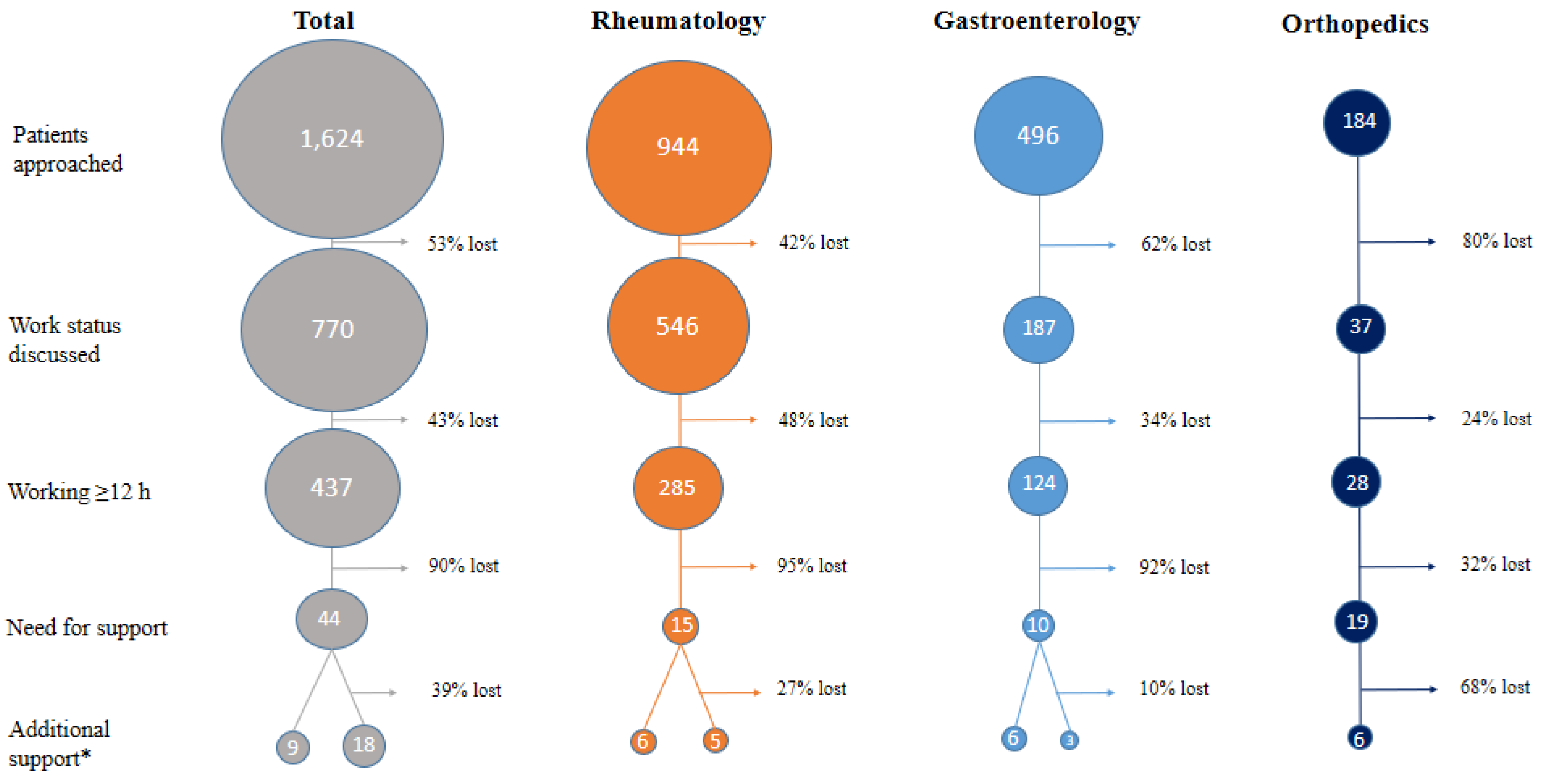

*Left balloon: low-complexity support (nurse); right balloon: high-complexity support (work participation clinic)

Supplementary Information
A mixed-methods process evaluation of the Maastricht work-related support intervention in clinical care for healthcare professionals in clinical care - Butink, M., Boonen, A., Boymans, T., Baadjou, V., Hazelzet, E., & De Rijk, A. - *Journal of Occupational Rehabilitation*

Online resource 3. Types of support provided at the work participation clinic

The types of support were counted in the patient records (electronic patient files) of patients who had their consultation at the work participation clinic. Support typically consisted of not just one type of support but rather a combination of several due to the complexity of the work-related problems.

Type of support	Counted in patient records
Discussing the impact of limited health on work	5
Improving coping	3
Starting a rehabilitation treatment	3
Starting or adjusting physiotherapy	2
Improving condition and strength	2
Starting Acceptance and Commitment Therapy	2
Referral to patient’s own occupational physician	1

Online resource 4. Evaluation by patients in the observational study that received low- or high-complexity support

	**Support by nurse (low-complexity)***	**Support by work participation clinic (high-complexity)**
Patients in observational study that received additional support (N=20)	N=13	N=7
Mean age (SD)	55.3 (8.6)	58.7 (4.8)
Female (%)	62%	57%
Advice received (%)	54%	57%
Number of support appointments**	N=13	N=7
1	10	3
2	1	0
4	0	2
5	1	1
6	0	1
7	1	0
Satisfaction with received support **	N=10	N=6
5	2	1
6	0	3
7	3	2
8	3	0
9	1	0
10	1	0
Helpfulness of the received support**	N=9	N=5
4	2	0
5	2	1
6	1	2
7	2	2
9	1	0
10	1	0
Formulation of a concrete plan (%)	0%	29%
Satisfaction with formulated plan†	NA	7.0 *(N=2)*
Performance to the plan†	NA	9.0 *(N=2)*
Motivation to adhere to the plan†	NA	6.5 *(N=2)*
Having work discussed in follow-up consultations with initial HCP‡	4.8 (2.4)	6.0 (2.8)
Recommending Maastricht WRS to other patients**	N=10	N=5
3	2	0
5	2	1
6	4	1
7	0	1
8	0	1
9	1	1
10	1	0

* Patients from Orthopedics could only be referred to the work participation clinic (no nurse for low-complexity support was available).
** Frequency; range 0-10 [0: not at all, to 10: very much], except the item 'number of support appointments' (ratio); outliers were excluded.
† Scale 0-10 [0: not at all, to 10: very much], expressed in mean. Only asked to participants who indicated having received a concrete plan (N=2).
‡ Scale 0-10 [0: not at all, to 10: very much], expressed in mean (SD).
